# Clinical Effect and Rotational Stability of TICL in the Treatment of Myopic Astigmatism

**DOI:** 10.1155/2020/3095302

**Published:** 2020-12-14

**Authors:** Manhui Zhu, Linling Zhu, Qiujian Zhu, Cailian Xu, Peng Yu, Haiyang Xiao, Ying Wang, You Yuan

**Affiliations:** ^1^Department of Ophthalmology, Lixiang Eye Hospital of Soochow University, Suzhou, Jiangsu, China; ^2^Department of Ophthalmology, Suzhou Municipal Hospital, The Affiliated Suzhou Hospital of Nanjing Medical University, Suzhou, Jiangsu, China

## Abstract

**Purpose:**

To investigate the clinical outcomes and possible risk factors associated with rotational stability after the implantation of a V4c toric implantable Collamer lens (TICL) for the correction of moderate to high myopic astigmatism.

**Methods:**

A total of 112 eyes of 66 patients with moderate to high myopic astigmatism underwent TICL implantation. All patients were followed up for more than 1 year. The uncorrected and best-corrected visual acuity (UCVA and BCVA), astigmatism and spherical equivalent, intraocular pressure, vault, endothelial cell morphometry, and rotation of the TICL axis were assessed at l day, 1 week, 1 month, 3 months, 6 months, and 12 months postoperatively. Postoperative rotation was defined as the angle between the intended axis and the achieved axis. Regression analysis was used to investigate the possible risk factors for TICL rotation postoperatively.

**Results:**

The mean efficacy index and safety index 12 months postoperatively were 1.03 ± 0.09 and 1.05 ± 0.10, respectively. All patients had the same or better visual acuity than preoperatively. The mean astigmatism value decreased from −1.86 ± 0.79 D preoperatively to −0.37 ± 0.35 D. The mean absolute axis deviation of the TICL at the last follow-up was 2.75 ± 2.04° (range, 0°∼11°). The mean manifest refraction spherical equivalent (MRSE) changed from -9.04 ± 2.67 D preoperatively to −0.67 ± 0.51 D postoperatively. The logistic regression demonstrated that the absolute degree of TICL rotation had a significant association with the fixation angle of the TICL and the size of the lens (*P*=0.003, *P*=0.026, resp.).

**Conclusion:**

The results of our study support that TICL implantation is safe, effective, and predictable in the treatment of moderate to high myopic astigmatism, with relatively good postoperative rotational stability.

## 1. Introduction

Myopia is a common cause of vision loss, especially high myopia. By one estimate, almost half of the global population will develop myopia, and 10% of the global population will develop high myopia [1]. Astigmatism is common in highly myopic eyes. Uncorrected astigmatism usually affects visual performance, leading to blurry, double vision. The toric implantable Collamer lens (TICL) is a posterior chamber phakic intraocular lens that is widely used for the correction of moderate to high myopic astigmatism [2]. Unlike laser ablation, it is not limited by high myopia, corneal thickness, or corneal curvature. TICL implantation achieves a more stable visual outcome and avoids the inherent risks of laser treatments [3].

TICL implantation has been reported to be predictable, safe, and effective in correcting moderate to high levels of astigmatism [3–5]. The accurate placement of a TICL is crucial to obtain satisfying visual outcomes. If the lens rotates after surgery, the cylinder correction effect is decreased. Therefore, both preoperative personalized design and precise postoperative measurement of rotational stability are important. The V4c TICL (STAAR Surgical Company, Monrovia, CA), with a central hole to allow the flow of aqueous humor without the need for an iridotomy, is increasingly being used in the correction of myopic astigmatism. Although some clinical studies have reported the efficacy and safety of the V4c TICL, the rotational stability has not been sufficiently researched. To our knowledge, only one study has reported factors influencing rotational stability, as identified by OPD-Scan III (NIDEK Co., Ltd., Gamagori, Japan) [6].

To better understand the mechanism of TICL rotation, the current study evaluated the clinical outcomes and rotational stability over a 12-month follow-up period after V4c TICL implantation and investigated various factors that might be related to rotation of the lens.

## 2. Materials and Methods

### 2.1. Patients

This study consisted of 112 consecutive eyes of 66 patients with moderate to high myopia from February to November 2018. All patients underwent V4c TICL (STAAR Surgical Company, Monrovia, CA) implantation by the same experienced surgeon. All the research and procedures in this study followed the tenets of the Declaration of Helsinki. Written informed consent was obtained from all patients. The study was approved by the Ethics Committee of Lixiang Eye Hospital of Soochow University (Soochow, China). All patients were followed up for more than 12 months after surgery.

The inclusion criteria were as follows: age 18 to 50 years; stable refraction for 2 years; presence of myopia with a manifest refraction spherical equivalent (MRSE) below −20.00 diopters (D); astigmatism between −0.75 and −5.00 D; and intraocular pressure (IOP) between 10 to 21 mmHg. Patients were excluded if the corneal endothelial cell density (ECD) was less than 2,000 cells/mm^2^ or the anterior chamber depth (ACD) was less than 2.7 mm. Patients who had a history of ocular surgery, trauma, cataracts, glaucoma, uveitis, or retinal detachment were also excluded.

### 2.2. Preoperative Examination

All patients underwent complete ophthalmic examinations, including E Standard logMAR uncorrected visual acuity (UCVA), best-corrected visual acuity (BCVA), manifest refraction, slit-lamp microscopy, dilated fundus examination, corneal endoscopy, noncontact tonometry, corneal topography by Pentacam, IOLMaster, optical coherence tomography (OCT), ultrasound biomicroscopy (UBM), and OPD-Scan III (NIDEK Co., Ltd., Gamagori, Japan) examination.

### 2.3. Surgical Procedure

The TICL power and size were chosen according to the formula supplied by the manufacturer. There are four lens sizes: 12.1, 12.6, 13.2, and 13.7 mm. The rotational placement of the lens is designed by the STAAR corporation and provided with an attached schematic diagram.

**Figure 1:**
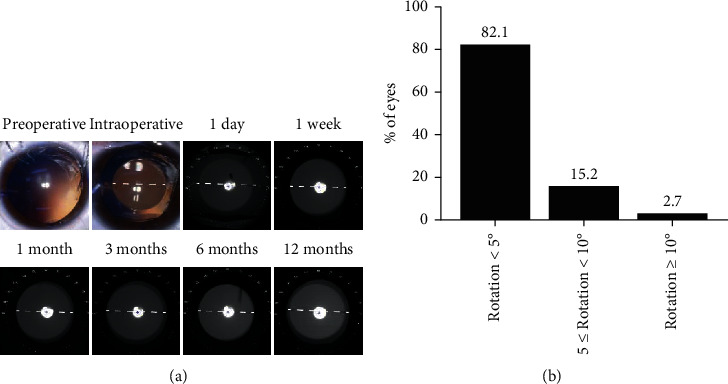
Presentation of the TICL axis after surgery. (a) Preoperative, intraoperative, and postoperative placement of the TICL. (b) Distribution of the TICL rotation at 12 months after surgery.

Before surgery, two points were marked using the slit-lamp while the patient was in a sitting position. Pupils were dilated 30 minutes before surgery using Mydrin-P (Santen Pharmaceutical Co., Ltd., Osaka, Japan). Topical anaesthesia with 0.5% Alcaine (Alcon Laboratories, Inc., Fort Worth, Texas, USA) was applied before the operation. After the injection of 1% sodium hyaluronate into the anterior chamber, the V4c TICL was then injected into the anterior chamber via a 2.8 mm temporal corneal incision using an injector cartridge and exactly aligned to the designed placement in the ciliary sulcus in the posterior chamber (Figure 1(a)). After that, the viscoelastic material was washed away using a syringe filled with balanced salt solution, and a miotic agent was then instilled. Postoperative topical eye drops included antibiotics and steroids.

Regular follow-up examinations were performed for each patient, including UCVA, BCVA, manifest refraction, slit-lamp examination, rotational stability, central vaulting (the distance from the posterior surface of the ICL at its center to the anterior surface of the crystalline lens), and IOP and corneal ECD examinations. Rotational stability (the disparity between the intended axis and the achieved axis) of the TICL was measured using the map obtained by OPD-Scan III (Figure 1(a)), as described in the report by Lee et al. [6]. To compensate for possible examination error, the measurements obtained on the day of surgery were used as a baseline. All eyes were examined after 1 day, 1 week, and 1, 3, 6, and 12 months. Each measurement was performed three times by an experienced technician, and the average value was used in the analysis.

### 2.4. Statistical Analysis

Statistical analysis was performed using SPSS software (version 23.0; IBM Corporation, Armonk, NY). Data are presented as the mean ± standard deviation. The comparison of visual acuity before and after the operation was performed by paired *t*-test. Repeated measures ANOVA was used to analyze differences among groups. Possible risk factors for TICL rotation, such as the preoperative spherical and cylindrical power, spherical equivalent refraction (SE), postoperative central vaulting, angle of the intended TICL axis off the horizontal meridian, ACD, white-to-white distance (WTW), and sulcus-to-sulcus distance (STS), were analyzed through an ordered logistic regression model. These variables were considered independent variables. The dependent variable was the absolute degree of rotation. A *P* value less than 0.05 was considered statistically significant.

## 3. Results

The mean patient age was 24.04 ± 5.03 years (range: 18 to 42 years). All eyes had preoperative myopia ranging from −1.25 to −17.00 D and a preoperative cylinder ranging from −0.50 to −4.00 D. Other baseline clinical characteristics are presented in Table 1. The results are expressed as the mean ± standard deviation (range). All surgeries were smooth, and no serious intraoperative complications were noted. No patient needed repositioning or explantation of the TICL.

### 3.1. Visual Acuity

**Figure 2:**
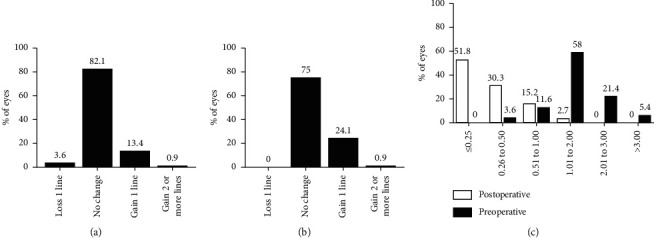
Visual acuity and refractive outcomes at 12 months after surgery. (a) Preoperative BCVA vs postoperative UCVA. (b) Preoperative BCVA vs postoperative BCVA. (c) Preoperative vs postoperative refractive cylinder.

After 12 months, 97.3% (109/112) of the eyes showed a UCVA of 1.0 or better, and all eyes had a UCVA of over 0.8, indicating the good efficacy of TICL implantation. The mean efficacy index (mean postoperative UCVA/mean preoperative BCVA) was 1.03 ± 0.09. Comparing the pre- and postoperative BCVA values, no patient had a BCVA lower than the preoperative value at the end of follow-up (*t* = 5.789, *P* < 0.01). Eighty-four eyes (75.0%) showed no change in the BCVA, 27 eyes (24.1%) gained 1 line, 1 eye gained 2 lines, and no eyes lost 1 or more lines (Figure 2(b)). The mean safety index (mean postoperative BCVA/mean preoperative BCVA) was 1.05 ± 0.10. A comparison of the preoperative BCVA values with the postoperative UCVA and BCVA values at different times is shown in Figure 2.

### 3.2. Refractive Outcomes

The mean manifest refractive cylinder decreased from −1.86 ± 0.79 D preoperatively to -0.37 ± 0.35 D at 12 months postoperatively (*t* = 18.994, *P* < 0.01). A total of 82.1% and 97.3% of the eyes were within −0.50 D and −1.00 D, respectively, indicating the good predictability of TICL implantation for astigmatism correction. The mean MRSE improved significantly from −9.04 ± 2.67 D preoperatively to −0.67 ± 0.51 D at 12 months postoperatively (*t* = 34.269, *P* < 0.01). The pre- and postoperative refractive results are shown in Figure 2(c).

### 3.3. Vault

The mean central vault measurement at 1 week, 1 month, 3 months, 6 months, and 12 months was 523.88 ± 135.96 mm, 524.82 ± 133.69 mm, 523.42 ± 137.40 mm, 522.08 ± 134.74 mm, and 521.50 ± 132.89 mm, respectively. There were no significant differences among the groups (*F* = 2.349, *P*=0.066) (Table 2).

### 3.4. Complications

No intraoperative or postoperative complications, such as pupillary block glaucoma or cataract formation, occurred during the follow-up period, and all TICL implantations were uneventful. The mean ECD at 12 months was 2772.81 ± 163.16 cells/mm^2^ (Table 2). The rate of corneal endothelial cell loss [(mean preoperative ECD − mean postoperative ECD) × 100%/mean preoperative ECD] at the end of follow-up was 1.45%. After 3 months, the IOP was stable (Table 2). Comparison of the pre- and postoperative IOP values showed no significant difference (*t* = 1.643, *P*=0.103).

### 3.5. Risk Factors for TICL Rotation

At 12 months postoperatively, the TICL showed clockwise misalignment in 57 (50.9%) of 112 eyes, counterclockwise misalignment in 47 (42.0%) eyes, and no misalignment in 8 (7.1%) eyes. The mean absolute value of the difference between the intended and achieved axes (rotational stability) was 2.75 ± 2.04° (range: 0 to 11°) at the last postoperative follow-up. The degree of rotation during different periods is presented in Table 2. There were no significant differences among the different periods (*F* = 1.093, *P*=0.352). The degree of rotation from the intended axis was <5° for 82.1% of the TICLs, <10° for 97.3% of TICLs, and ≥10° for two (2.7%) TICLs (Figure 1(b)). No eyes underwent a second procedure to reposition the TICL.

To investigate the risk factors for postoperative TICL rotation, binary logistic regression analysis was performed. The dependent variable was the absolute degree of rotation divided into two categories (1: rotation <5°, 2: rotation ≥5°). Risk factors such as the preoperative spherical and cylindrical degree, MRSE, postoperative central vaulting, angle of intended TICL axis off the horizontal meridian, ACD, WTW distance, and STS distance were considered independent variables. The results are presented in Table 3. The degree of TICL rotation showed a significant association with the intraoperative fixation angle provided by the manufacturer's instructions and the size of the TICL (*P*=0.003, *P*=0.026, resp.). Then, an ordered, multinomial regression model was built to analyze the relation between the angle of TICL rotation and the absolute degree of the intended TICL axis off the horizontal meridian. The dependent variable was divided into three categories (1: rotation <5°, 2 : 5 ≤rotation <10°, 3: rotation ≥10°), and the independent variable was also divided into three categories (1: rotation ≥10°, 2 : 5 ≤rotation <10°, 3: rotation <5°). The results are shown in Table 4. Eyes with an intraoperative fixation angle of 5° or more were more likely to show rotation than eyes with a fixation angle of less than 5°.

## 4. Discussion

Currently, the TICL is widely used for the correction of myopic astigmatism. It has been reported as a predictable, safe, and effective treatment in correcting astigmatism [2, 4, 7]. In this study, we analyzed the visual acuity, refractive outcomes, and rotational stability after implantation of the V4c TICL in 112 eyes of 66 patients. The results show that the V4c TICL performed well in terms of efficacy, safety, and rotational stability in patients after surgery. Although there have been several articles reaching the same conclusion, our study was the first to investigate the risk factors for TICL rotation by an ordinal multinomial regression approach.

In our study, visual acuity after 12 months was satisfactory. The mean efficacy index and safety index were 1.03 ± 0.09 and 1.05 ± 0.10, respectively. No patient had a UCVA under 0.8, and no patient had a BCVA lower than the preoperative value at the end of follow-up. A total of 110 of 112 eyes (82.1%) had a refractive cylinder within −0.50 D, indicating good predictability of TICL implantation for astigmatism correction.

The V4c TICL has a 360 *μ*m port in the center of the optical zone and two perioptic holes. The holes allow natural flow of aqueous humor, avoiding the need for Nd : YAG laser peripheral iridotomy (PI) before ICL implantation. Furthermore, the V4c ICL was stored in BSS and did not enlarge after implantation. It has been reported that immediate fixation in the ciliary sulcus after implantation leads to better rotational stability [2]. Bhandari et al. [8] found that the implantation of an ICL with a central hole resulted in minimal postoperative IOP fluctuations without additional PI. ICLs with holes may have advantages over conventional ICLs in terms of corneal endothelial cell loss [9].

The crucial factor in correcting cylinder with TICL implantation appears to be the stability of the axis. It is well accepted that a rotation of the intraocular lens 10° away from the intended implantation axis increases refraction and decreases optical performance [10–12]. A rotation of 30° has no effect on correcting astigmatism [13]. The internal OPD map obtained by OPD-Scan III was used to assess the rotational stability. It has been reported to be more accurate and have a lower standard deviation than slit-lamp digital imaging or slit-lamp assessments [7, 14]. In the current study, the mean rotation at 12 months was 2.75 ± 2.04°, which is similar to the results in previous studies by Damho et al. (2.4 ± 3.8°) [7] and Ayman et al. (2.68 ± 2.11°) [14]. The maximum TICL rotation in eyes with rotation occurred at 6 months, but there was no statistically significant difference in rotation among the time points.

To investigate the risk factors for postoperative TICL rotation, binary logistic regression analysis was performed. We found that the absolute degree of the intended TICL axis off the horizontal meridian (the intraoperative fixation angle) and the size of the TICL were significantly correlated with postoperative rotation. It is certain that an undersized TICL results in a low vault and rotates more easily, while an oversized TICL results in a high vault and does not easily rotate [15]. Presently, there are 4 models to choose from. In the clinic, an intraocular lens with a larger size can be selected to increase the stability if the depth of the anterior chamber is sufficient. An ordinal multinomial regression was used to analyze the role of the fixation angle in TICL rotation. The results show that eyes with a fixation angle of more than 10 degrees were 6.02 times more likely to show larger degrees of rotation than eyes with an angle of less than 5 degrees. This is similar to the results of a previous study by Mori et al. [16], which demonstrated that eyes with a fixation angle of 5° or more were 5.6 times more likely to show rotation than eyes with an angle of less than 5°. We also found that eyes with a fixation angle of 5 to 10 degrees were 4.24 times more likely to show larger degrees of rotation than eyes with an angle of less than 5 degrees (Table 4). Thus, it is better to control the fixation angle to within 5 degrees. In our study, the largest fixation angle was 21 degrees, and the largest postoperative degree of rotation was 11 degrees.

In a report by Park et al. [5], TICL rotation was not correlated with the angle of lens fixation, a finding that conflicts with our results. Hun et al. also demonstrated that no explanatory variable relevant to the absolute degree of rotation was discovered [6]. A possible explanation may be that the angle of fixation and degree of rotation of the TICL in our study are different from those in their studies.

A previous study [15] showed that TICL rotation had a significant correlation with the vault, while in our study, we did not find such a correlation. One reason for the lack of statistical significance may be the lack of cases with a very low or high vault. A very low vault may cause clinically significant postoperative TICL rotation. Further study with different vaults is needed to fully understand the relationship between the postoperative vault and rotation in V4c TICL implantation.

In Sheng et al.'s study [15], a significant correlation between the absolute degrees of rotation and the position of TICL footplate fixation was observed. Ideally, the ICL footplates should be placed in the ciliary sulcus. However, they hypothesized that footplates resting on the ciliary body may provide more stability than those resting in the ciliary sulcus. The location of the TICL footplates is an important factor determining the ICL vault, which could be affected by improper ICL size and structural abnormalities of the ciliary sulcus, such as ciliary body cysts or iris tumours [17]. One limitation of this study is that we did not analyze the association between the ICL footplate position and degree of rotation. However, we investigated the relation between TICL rotational stability and ciliary body cysts; no statistically significant relationship (*P*=0.308) was noted between them (data not shown).

In conclusion, the V4c TICL is effective in correcting astigmatism and improving visual acuity. Generally, postoperative rotational stability of the TICL was good, without the need for repositioning in our cases. We performed an ordered, multinomial regression analysis of possible risk factors for postoperative TICL rotation. Further studies with a larger sample size and a longer follow-up period are needed to explore more risk factors and the underlying mechanism.

## Figures and Tables

**Table 1:** Preoperative patient clinical data.

Characteristic	Value
*Age (y)*	
Mean ± SD	24.04 ± 5.03
Range	18, 42

*Sex (n)*	
Male	42
Female	70

*Manifest refractive sphere (D)*	
Mean ± SD	−8.10 ± 2.65
Range	−1.25, −17.00

*Manifest refractive cylinder (D)*	
Mean ± SD	−1.86 ± 0.79
Range	−0.50, −4.00

*Manifest SE (D)*	
Mean ± SD	−9.04 ± 2.67
Range	−3.13, −18.50

*Axial length (mm)*	
Mean ± SD	27.12 ± 1.34
Range	23.93, 31.89

*Intraocular pressure (mmHg)*	
Mean ± SD	14.73 ± 2.58
Range	10.0, 20.8

*Anterior chamber depth (ACD) (mm)*	
Mean ± SD	3.24 ± 0.23
Range	2.70, 3.89

*White-to-white distance (WTW) (mm)*	
Mean ± SD	11.61 ± 0.39
Range	10.70, 12.40

*Sulcus-to-sulcus distance (STS) (mm)*	
Mean ± SD	11.89 ± 0.47
Range	10.46, 12.98

*ICL model (n)*	
Size 12.1	14
Size 12.6	34
Size 13.2	60
Size 13.7	4

D = diopters; SE = spherical equivalent; ICL = implantable Collamer lens.

**Table 2:** Postoperative characteristics of patients after TICL implantation.

Characteristic	1 week	1 month	3 months	6 months	12 months
Central vaulting (mm)	523.88 ± 135.96	524.82 ± 133.69	523.42 ± 137.40	522.08 ± 134.74	521.50 ± 132.89
Mean endothelial cell density (cells/mm^2^)	2764.60 ± 193.90	2762.46 ± 196.37	2746.67 ± 162.65	2756.77 ± 157.29	2772.81 ± 163.16
Intraocular pressure (mmHg)	15.2 ± 2.2	14.8 ± 2.0	13.9 ± 2.8	13.9 ± 2.2	14.3 ± 2.4
Absolute degree of rotation (°)	2.49 ± 1.67	2.63 ± 1.78	2.72 ± 2.05	2.76 ± 2.09	2.75 ± 2.04

**Table 3:** Binary logistic regression analysis to evaluate the risk factors for TICL rotation at 12 months after surgery.

Variable	Regression coefficient (*β*)	*P* value
Age (y)	0.013	0.790
Sex (n)	0.216	0.691
Preoperative sphere (D)	−0.036	0.706
Preoperative cylinder (D)	0.164	0.630
Intraoperative fixation angle (°)	0.141	0.003^*∗*^
Mean central vault (mm)	0.001	0.776
Preoperative ACD (mm)	−0.587	0.607
Preoperative WTW (mm)	−1.191	0.076
Preoperative horizontal STS (mm)	−0.456	0.415
Preoperative vertical STS (mm)	−0.552	0.291
Axial length (mm)	−0.292	0.169
ICL diameter (mm)	−1.344	0.026^*∗*^

ACD = anterior chamber depth; WTW = white-to-white distance; STS = sulcus-to-sulcus distance.

**Table 4:** Ordered, multinomial regression to analyze the association between postoperative TICL rotation and intraoperative fixation angle.

Category	Mean rotation (°)	Rotation <5°	5° ≤Rotation <10°	Rotation ≥10°	OR
Fixation angle ≥10°	3.63 ± 1.63	9	6	1	6.02
5° ≤fixation angle ≤10°	3.24 ± 2.20	19	5	1	4.24
Fixation angle ≤5°	2.27 ± 1.81	66	5	0	—

OR = odds ratio.

## Data Availability

The data that support the findings of this study are available from the corresponding author upon reasonable request.
